# Mutation Accumulation May Only Be a Minor Force in Shaping Life-History Traits, Even when Reproduction Is Sexual

**DOI:** 10.1371/journal.pone.0048302

**Published:** 2012-10-31

**Authors:** Maciej Jan Dańko, Jan Kozłowski

**Affiliations:** 1 Max Planck Institute for Demographic Research, Rostock, Germany; 2 Institute of Environmental Sciences, Jagiellonian University, Kraków, Poland; Centers for Disease Control and Prevention, United States of America

## Abstract

In a previous theoretical study we investigated whether adaptive or non-adaptive processes are more important in the evolution of senescence. We built a model that combined both processes and found that mutation accumulation is important only at those ages where mortality has a negligible impact on fitness. This model, however, was limited to haploid organisms. Here we extend our model by introducing diploidy and sexual reproduction. We assume that only recessive (mutated) homozygotes experience detrimental effects. Our results corroborate our previous conclusions, confirming that life histories are largely determined by adaptive processes. We also found that the equilibrium frequencies of mutated alleles are at higher values than in haploid model, because mutations in heterozygotes are hidden for directional selection. Nevertheless, the equilibrium frequencies of recessive homozygotes that make mutations visible to selection are very similar to the equilibrium frequencies of these alleles in our haploid model. Diploidy and sexual reproduction with recombination slows down approaching selection-mutation balance.

## Introduction

The evolution of senescence can be explained on the basis of two classes of theory: a non-adaptive theory (mutation accumulation [1]) and adaptive theories (antagonistic pleiotropy [2], disposable soma theory [3]). These approaches are not mutually exclusive, but there is still debate as to which of them is more important (e.g., [4], [5], [6], [7], [8] and [9], reviewed in [10]). In a previous article published in PLoS ONE [10] we presented a model that addressed this question. Our main contribution was to combine adaptive and non-adaptive processes in a single model. Our major finding was that even if more age-specific mutations accumulate at higher ages, this accumulation has only minor effects on shaping life-histories represented by age at maturity.

The main weakness of that model, however, was its assumption of asexual haploid reproduction. Sexual reproduction and diploidy are features of many higher organisms. Sexual reproduction, with recombination occurring at production of gametes, is thought to help avoid Muller’s Ratchet [11], a decrease of fitness from generation to generation driven by mutation accumulation. From the perspective of mutation accumulation, sexual reproduction and diploidy could theoretically lead to a mutation-selection balance with a higher frequency of deleterious mutations.

Our assumption of asexual haploid reproduction led to appropriate questions regarding the applicability of our results to sexual diploid species; here we explicitly address these concerns. We extend the previous approach by adding diploidy and sexual reproduction into our model. We assume that only recessive (mutated) homozygotes experience detrimental effects. We hypothesize that diploidy and sexual reproduction can increase the frequency of age-specific mutations, occurring in both homo- and heterozygotes. However, we also expect that the phenotypic effect of mutations predicted by the diploid model (based on the frequency of recessive homozygotes for mutations effecting each age) should be distributed across the ages identically or very similarly to those predicted by the haploid model (based on simple allele frequencies). Thus sexual reproduction should not alter the main conclusion of the previous paper: mutation accumulation should have a minor effect on early life history traits, such as age at maturity.

**Figure 1:**
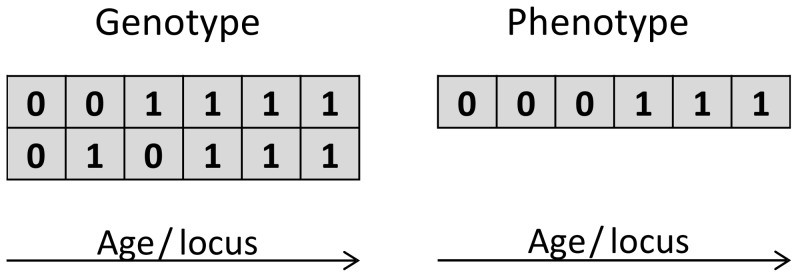
The effect of recessive mutations in two chromatids on the phenotype. Zeros in chromatids represent not mutated genes, and zeros in phenotype represent no phenotypic effect.

**Table 1:** Calculating frequencies of different genotypes in different loci (simplified example for the 2-locus case).

				Contribution to genotype frequency			
Genotype				Recessive homozygote		Heterozygote	
*g*	Chromatid	Chromatid	Freq.	Locus	Locus	Locus	Locus
	1	2		1	2	1	2
1	0 0	0 0	*f*_1_	0	0	0	0
2	0 0	0 1	*f*_2_	0	0	0	*f*_2_
3	0 0	1 0	*f*_3_	0	0	*f*_3_	0
4	0 0	1 1	*f*_4_	0	0	*f*_4_	*f*_4_
5	0 1	0 0	*f*_5_	0	0	0	*f*_5_
6	0 1	0 1	*f*_6_	0	*f*_6_	0	0
7	0 1	1 0	*f*_7_	0	0	*f*_7_	*f*_7_
8	0 1	1 1	*f*_8_	0	*f*_8_	*f*_8_	0
9	1 0	0 0	*f*_9_	0	0	*f*_9_	0
10	1 0	0 1	*f*_10_	0	0	*f*_10_	*f*_10_
11	1 0	1 0	*f*_11_	*f*_11_	0	0	0
12	1 0	1 1	*f*_12_	*f*_12_	0	0	*f*_12_
13	1 1	0 0	*f*_13_	0	0	*f*_13_	*f*_13_
14	1 1	0 1	*f*_14_	0	*f*_14_	*f*_14_	0
15	1 1	1 0	*f*_15_	*f*_15_	0	0	*f*_15_
16	1 1	1 1	*f*_16_	*f*_16_	*f*_16_	0	0
Genotype frequency at different loci:				P_aa_(1) = Σ(…)	P_aa_(2) = Σ(…)	P_Aa_(1) = Σ(…)	P_Aa_(2) = Σ(…)

*P_aa_* and *P_Aa_* are frequencies of recessive (mutated) homozygotes and heterozygotes respectively and *f_g_* is frequency of a genotype with number *g*. Each chromatid is represented as a bit string, where 1 denotes a mutated locus and 0 a non-mutated one. A genotype contributes to the frequency of recessive homozygote at locus *n* if both chromatids have mutation at position *n*. Similarly, a heterozygote at locus *n* is contributed by the genotype if there is one and only one mutation in both chromatids at position *n*. The frequency of mutated alleles is calculated in each locus according to the equation *p*(*n*) = *P_aa_* (*n*) + 0.5 *P_Aa_* (*n*).

## Methods

The model presented in this article is a modification of the one proposed by Dańko et al. [10]. In the next sections we briefly present the major assumptions of the previous model and then we give details about the modifications.

The existence of age specific-gene effects are introduced into the model by adopting the idea of Penna’s bit-strings ([12], reviewed in [13]). Under this approach each genotype is represented as a vector of 0’s and 1’s, where 1 denotes a mutated gene. The mutated gene represented by the *n*th bit-gene in the bit string takes effect at the beginning of the *n*th age interval. Once activated, the action of a gene persists until the end of life. A genotype with no mutations at any loci experiences constant mortality, equal to the constant background death rate 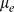. We assume that a mutated gene increases mortality additively by a constant 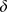 from that age onwards. For simplicity, the effect of mutations on reproduction rate is not investigated here, because it was discussed in the previous paper. To determine age-interval length we need to limit the lifespan. The maximum age 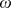 in the population (calculated for the same parameters set) is set to the age when remaining reproduction falls below 0.0001 of total reproduction for the non-mutated genotype, since ages beyond that point do not significantly alter fitness for any genotype.

**Figure 2:**
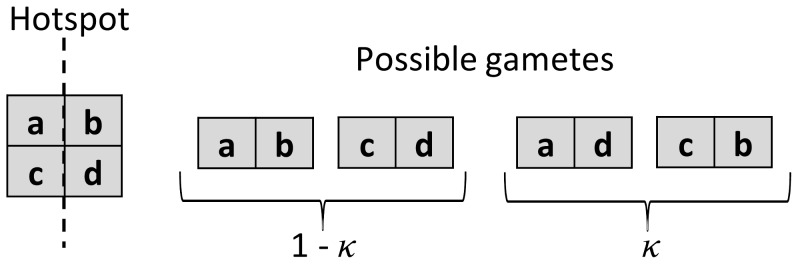
Crossing-over in the model. It is symmetrical and takes place in the middle of the chromosome. a, b, c and d represent bit-strings of equal lengths, *κ* is the probability of crossing-over.

**Table 2:** Calculating new frequencies of gametes (simplified example for the 2-locus case).

Genotype			No crossing-over		Crossing-over		Conditional frequencies			
Chromatid	Chromatid	Freq.	Gamete	Gamete	Gamete	Gamete	Gamete	Gamete	Gamete	Gamete
1	2		1	2	1	2	1	2	3	4
0 0	0 0	*f*_1_	**0 0**	**0 0**	**0 0**	**0 0**	**(1−** ***κ*) *f*_1_**	**(1−** ***κ*) *f*_1_**	***κ f*** **_1_**	***κ f*** **_1_**
0 0	0 1	*f*_2_	**0 0**	0 1	0 1	**0 0**	**(1−** ***κ*) *f*_2_**	(1−*κ*) *f*_2_	*κ f*_2_	***κ f*** **_2_**
0 0	1 0	*f*_3_	**0 0**	1 0	**0 0**	1 0	**(1−** ***κ*) *f*_3_**	(1−*κ*) *f*_3_	***κ f*** **_3_**	*κ f*_3_
0 0	1 1	*f*_4_	**0 0**	1 1	0 1	1 0	**(1−** ***κ*) *f*_4_**	(1−*κ*) *f*_4_	*κ f*_4_	*κ f*_4_
0 1	0 0	*f*_5_	0 1	**0 0**	**0 0**	0 1	(1−*κ*) *f*_5_	**(1−** ***κ*) *f*_5_**	***κ f*** **_5_**	*κ f*_5_
0 1	0 1	*f*_6_	0 1	0 1	0 1	0 1	(1−*κ*) *f*_6_	(1−*κ*) *f*_6_	*κ f*_6_	*κ f*_6_
0 1	1 0	*f*_7_	0 1	1 0	**0 0**	1 1	(1−*κ*) *f*_7_	(1−*κ*) *f*_7_	***κ f*** **_7_**	*κ f*_7_
0 1	1 1	*f*_8_	0 1	1 1	0 1	1 1	(1−*κ*) *f*_8_	(1−*κ*) *f*_8_	*κ f*_8_	*κ f*_8_
1 0	0 0	*f*_9_	1 0	**0 0**	1 0	**0 0**	(1−*κ*) *f*_9_	**(1−** ***κ*) *f*_9_**	*κ f*_9_	***κ f*** **_9_**
1 0	0 1	*f*_10_	1 0	0 1	1 1	**0 0**	(1−*κ*) *f*_10_	(1−*κ*) *f*_10_	*κ f*_10_	***κ f*** **_10_**
1 0	1 0	*f*_11_	1 0	1 0	1 0	1 0	(1−*κ*) *f*_11_	(1−*κ*) *f*_11_	*κ f*_11_	*κ f*_11_
1 0	1 1	*f*_12_	1 0	1 1	1 1	1 0	(1−*κ*) *f*_12_	(1−*κ*) *f*_12_	*κ f*_12_	*κ f*_12_
1 1	0 0	*f*_13_	1 1	**0 0**	1 0	0 1	(1−*κ*) *f*_13_	**(1−** ***κ*) *f*_13_**	*κ f*_13_	*κ f*_13_
1 1	0 1	*f*_14_	1 1	0 1	1 1	0 1	(1−*κ*) *f*_14_	(1−*κ*) *f*_14_	*κ f*_14_	*κ f*_14_
1 1	1 0	*f*_15_	1 1	1 0	1 0	1 1	(1−*κ*) *f*_15_	(1−*κ*) *f*_15_	*κ f*_15_	*κ f*_15_
1 1	1 1	*f*_16_	1 1	1 1	1 1	1 1	(1−*κ*) *f*_16_	(1−*κ*) *f*_16_	*κ f*_16_	*κ f*_16_

Columns 1–2: possible genotypes, two columns represent first and second chromatid. Third column: frequencies of different genotypes after selection. Columns 4–7: possible gametes generated by one genotype when there is no crossing-over (probability: 1−*κ*) and when the crossing-over takes place (probability: *κ*). Each genotype and gamete is represented as a bit string, where 1 denotes mutated locus and 0 not mutated one. Last four columns: conditional frequencies of occurrence of different gametes generated by one genotype. There are four different types of gametes: {00}, {01}, {10} and {11}. The frequency of each type of gametes is calculated as a sum of all conditional frequencies for a specified gamete and divided by 2, e.g., for gamete {00} (bold and underline) : F_00_ = [(1−*κ*)*f*_1_ + (1−*κ*)*f*_1_ + *κ f*_1_ + *κ f*_1_ + (1−*κ*)*f*_2_ + *κ f*_2_ + (1−*κ*)*f*_3_ + *κ f*_3_ + (1−*κ*)*f*_4_ + (1−*κ*)*f*_5_ + *κ f*_5_ + *κ f*_7_ + (1−*κ*)*f*_9_ + *κ f*_9_ + *κ f*_10_ + (1−*κ*)*f*_13_] / 2.

**Table 3:** Random mating and calculation of frequency of genotypes after sexual reproduction (simplified example for the 2-locus case).

	Gamete B				Gamete B			
Gamete A	0 0	0 1	1 0	1 1	0 0	0 1	1 0	1 1
**0 0**	00 00	00 01	00 10	00 11	F_00_*F_00_	F_00_*F_01_	F_00_*F_10_	F_00_*F_11_
**0 1**	01 00	01 01	01 10	01 11	F_01_*F_00_	F_01_*F_01_	F_01_*F_10_	F_01_*F_11_
**1 0**	10 00	10 01	10 10	10 11	F_10_*F_00_	F_10_*F_01_	F_10_*F_10_	F_10_*F_11_
**1 1**	11 00	11 01	11 10	11 11	F_11_*F_00_	F_11_*F_01_	F_11_*F_10_	F_11_*F_11_
	**Genotype**				**Frequency**			

The frequencies of gametes are denoted as F and the method of their calculations is shown in Table 2.

For each genotype we calculate the optimal age at maturity that maximizes our measure of fitness, which is lifetime expected allocation to reproduction. Each generation is simulated by an iteration of two stages: (i) Selection, when the frequencies of genotypes change proportionally to the calculated fitness, and (ii) mutations, when the frequencies of genotypes change due to mutations in each locus. The algorithm stops once mutation-selection equilibrium is reached. The population has non-overlapping generations and abundance of genotypes is defined in terms of a gene frequency, rather than a number of individuals. We assume constant background mortality. For further details please see [10].

**Figure 3:**
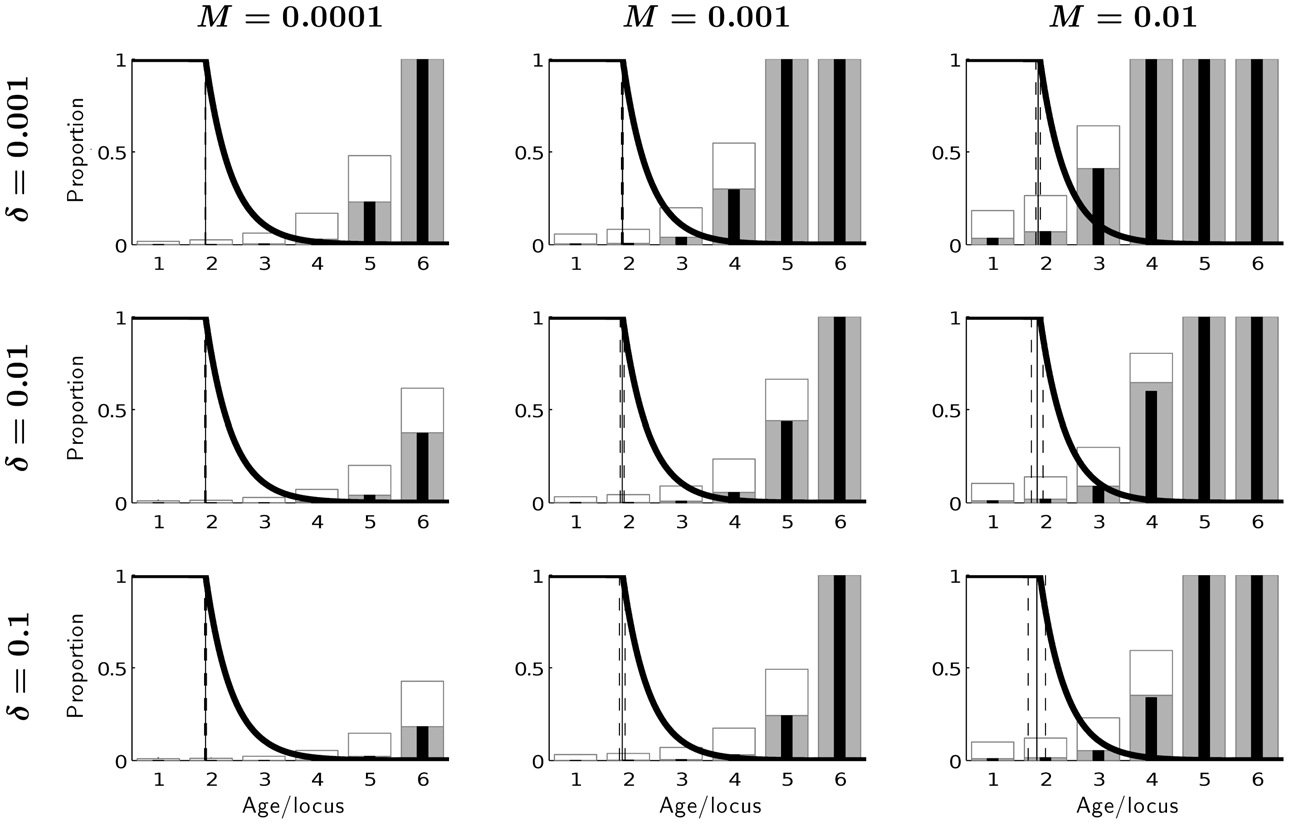
Equlibrium frequencies of mutations at different loci for background mortality *µ_e_* = 0.01 under different mutation rates *M* and different effects of mutations on mortality *δ*. Thin black bars: equilibrium frequencies of mutations for the asexual model, thick grey bars: equilibrium frequencies of recessive mutated homozygotes for the sexual model (see Fig. 1, Phenotype), empty bars: equilibrium frequencies of recessive mutated alleles at different loci for the sexual model (see Tab. 1). The vertical lines represent mean optimal age at maturity (solid) with standard deviation (dashed). The thick solid line captures the fraction of remaining reproduction, which is similar to Hamilton’s force of selection [16].

**Figure 4:**
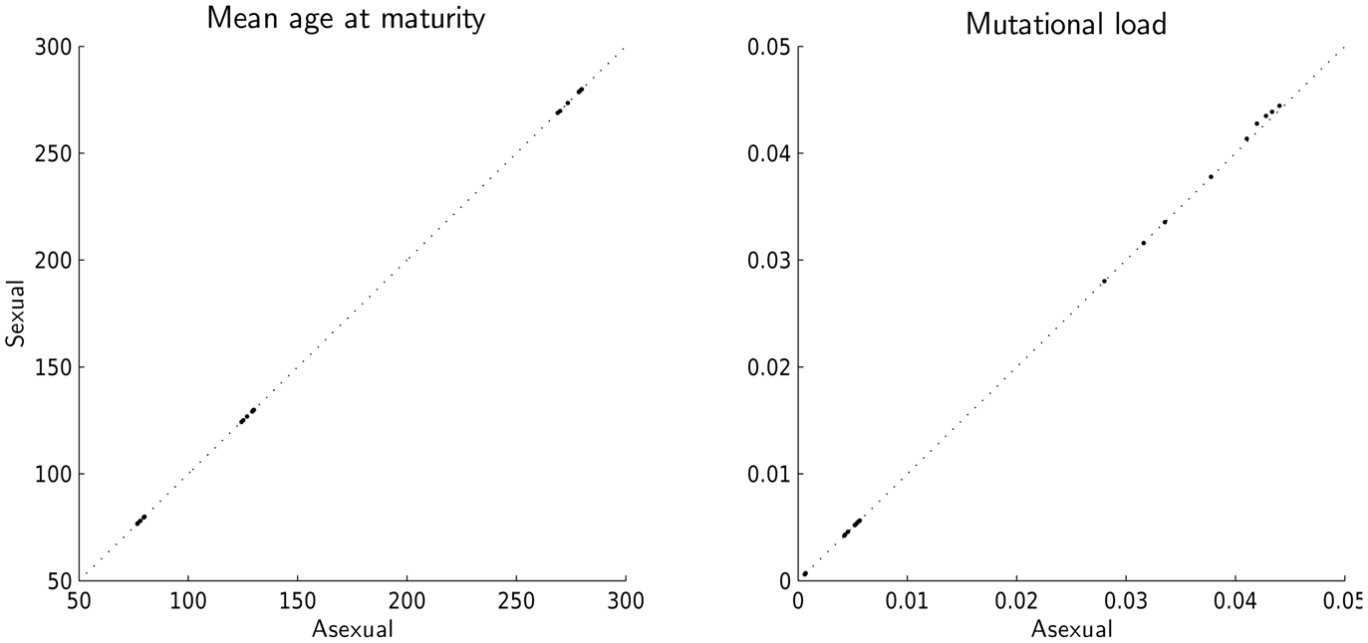
Comparison of mean optimal age at maturity and mutational load for different sets of parameters in the haploid (Asexual) model and diploid (Sexual) model with sexual reproduction. Parameter values tested in different combinations: mutation rates per locus, *M* = 0.0001, 0.001 and 0.01; effects of mutations on mortality, 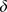 = 0.001, 0.01 and 0.1; levels of background death rates, 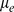 = 0.01, 0.02 and 0.03. Diagonal line represents situation when parameter values for haploid and diploid models are the same.

**Figure 5:**
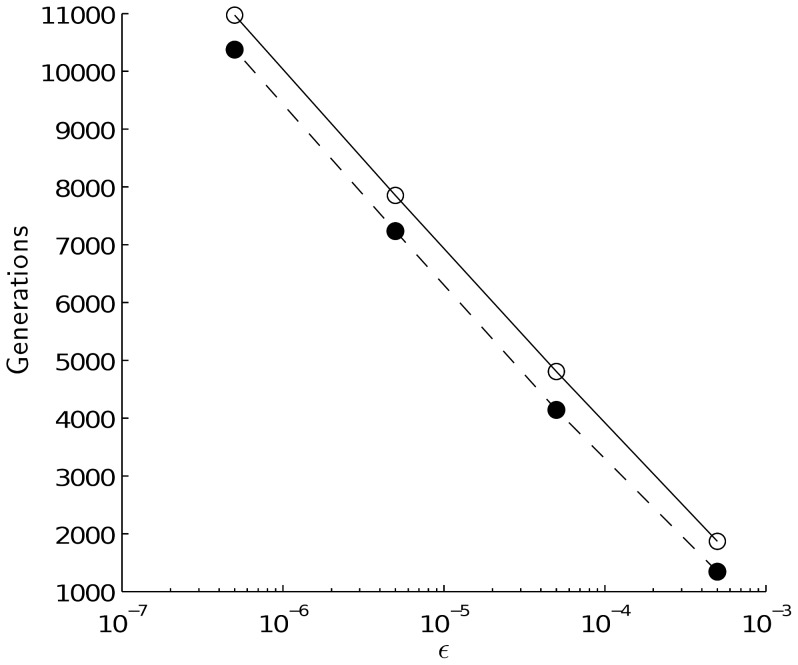
Number of generations required to approach mutation-selection equilibrium with different precision *ε*. Asexual population: dashed line, sexual population: solid line. The calculations started with one non-mutated genotype under parameters 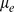 = 0.01, *M* = 0.001 and 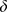 = 0.01.

In the previous model we assumed 10 independent age-specific loci, which was demonstrated to be the best compromise between model precision and computing demands. Here the number of loci is limited to 6 by computational demands. We believe that the lower number of loci does not compromise the explanatory value of the model, because in this model we are mainly interested in differences between sexual and asexual reproduction.

As has been stated previously, the model is extended by the addition of diploidy and sexual reproduction. Diploidy is introduced by adding an extra chromatid (Fig. 1). Each chromatid can mutate independently. We assume that only recessive homozygotes express negative effects. In this case phenotypic effects are only present if both genes on the same locus are mutated. For the whole population the phenotypic frequency and the genotypic frequency can be calculated using analogical methods to those presented in Table 1 of our previous paper [10] (see also Table 1 of this paper).

In the algorithm, sexual reproduction is placed between selection of the genotypes and mutations. As in our previous model, all calculations are based on genotype frequencies. At the beginning, the frequencies of all possible gametes are calculated. The routine for calculation of gametes frequencies is exemplified (simplified 2-locus case not used in real calculations) in Table 2. These frequencies may be affected by chromosomal crossing-over. The process has only one crossing-over point in the middle of each chromatid. The resulting recombination may increase the diversity of gametes that come from each parental genotype. The mechanism of crossing-over is presented in Fig. 2. Once the frequencies of gametes are calculated then gametes mate randomly and new distributions of genotypes can be calculated (see Table 3).

The cycle of selection, sexual reproduction and mutations is repeated until equilibrium frequencies of genotypes are reached. The equilibrium condition is fulfilled when the sum of absolute differences between two distributions of genotypes in two consecutive generations is lower than *ε = *0.000001. We test also different values of *ε* to check the speed of the evolutionary process for comparing the diploid to the haploid model.

## Results and Discussion

We test our model under different combinations of per locus mutation probabilities, *M* = 0.0001, 0.001 and 0.01, different effects of mutations on mortality, 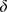 = 0.001, 0.01 and 0.1, different levels of background death rates, 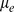 = 0.01, 0.02 and 0.03 and different crossing-over probabilities.

The influence of mutation accumulation on shaping life history patterns, exemplified by optimal age at maturity *τ* across equilibrated genotypes, can generally be neglected if we take into account its average value. Nevertheless, we observe a slight increase in variation of this trait with mutation rate (*M*) because the distribution of genotypes has greater variance and genotypes differ in *τ* (Fig. 3). The strength of the mutation effect on mortality 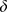 also affects the variance of *τ*, because differences between optimal ages at maturity become larger. Furthermore, even genotypes differing only in one locus may differ greatly in *τ,* especially if the effect of this locus occurs early in life. Even a small fraction of such genotypes has a strong effect on the variance of optimal age at maturity, especially at high 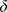. This fraction is greater when mutation pressure is high, which explains the role of *M*.

Phenotypic frequencies of mutations affecting different ages, are generally neglible at ages that contribute significantly to evolutionary fitness regardless of whether the population is sexual diploid or asexual haploid. These frequencies are similar between models under different combinations of mutation rates *M* and different magnitudes of effect of mutations on mortality 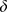 (Fig. 3), and also for different levels of background mortality (not shown). For asexual haploids phenotypic frequencies equal genotypic frequencies. For diploid sexuals harmful mutations appear in both recesive homozygotes with phenotypic effect and heterozygotes without phenotypic effect. Figure 3 shows clearly that the mutations are present at relatively high frequency even at ages relevant to fitness. However, mutated phenotypes appear at the same frequencies as in asexual haploid model, which means that sexual reproduction does not change mutational load (decrease of fitness with respect to non-mutated case [14]).

For both sexual and asexual cases mean optimal age at maturity and mutational load reveal a very weak influence of mutation accumulation (not shown for the diploid model, because it is roughly the same as in the haploid one). Furthermore, both measures are very similar for sexual and asexual reproduction (Fig. 4). Interestingly, the probability of crossing-over has neglible influence on the results. It seems that under an assumption of an infinite population size crossing-over is not an important process and cannot aid selection in removing mutated genes. We believe that in small finite populations, where drift plays a role, this effect may be more important.

Diploidy decreases the rate at which population approaches selection-mutation equlibrium (Fig. 5). This is obvious, because mutations hidden in heterozygotes are invisible to selection.

Finally we observe similar effect of extrinsic mortality on qualitative patterns of mutation accumulation as in haploid model (not shown), indicating that background mortality determines the “pace” of mutation accumulation, but it negligibly affects its “shape” ([10], see also [15] for explanation of terms “pace” and “shape”).

In conclusion, mutation acumulation is a minor force in shaping life history traits even if diploidy and sexual reproduction are introduced. Mutations manifest their effects only in homozygotes and are under strong directional selection only at young ages. The frequency of mutated homozygotes may be high only for genes with effect at age classes having negligible effect on fitness. While sexual reproduction, strongly increasing genetic variation, can have many effects on both the process and outcome of selection in general, it does not affect mutation accumulation effects on the life history. Thus, all previous results achieved with the simple haploid model in [10] must hold also under diploidy with sexual reproduction. These include: (i) no significant effect of mutation accumulation on mean optimal age at maturity, (ii) low mutational load and (iii) significant mutation accumulation effects observed only at ages with very low remaining reproduction and very low survivability. We conclude that while it is important to examine whether theoretical results achieved in a haploid model can be applied to a diploid sexual population, in this instance there is no appreciable difference.
